# Molecular and seroepidemiology of SARS-CoV-2 among influenza-like illness and severe Acute respiratory illness cases in selected health facilities in Ethiopia

**DOI:** 10.3389/fpubh.2025.1714899

**Published:** 2026-01-26

**Authors:** Adamu Tayachew, Dawit Assefa Arimide, Wolde Shure, Dejenie Shiferaw Teklu, Ayele Gebeyehu, Tsegaye Berkesa, Gizaw Teka, Mandefro Kebede, Mesfin Wossen, Melkamu Abte, Mesay Hailu, Zelalem Mekuria, Nega Berhe, Patrik Medstrand, Nigatu Kebede

**Affiliations:** 1 Aklilu Lemma Institute of Pathobiology, Addis Ababa University, Addis Ababa, Ethiopia; 2 Ethiopian Public Health Institute, Addis Ababa, Ethiopia; 3 Translational Medicine, Clinical Virology, Lund University, Malmö, Sweden; 4 Department of Veterinary Preventive Medicine, Ohio State University College of Veterinary Medicine, Columbus, OH, United States; 5 Science for Life Laboratory, Lund University, Lund, Sweden; 6 Clinical Microbiology, Infection Control and Prevention, Laboratory Medicine, Lund, Sweden

**Keywords:** ELISA, epidemiology, Ethiopia, post-pandemic, SARS-CoV-2, whole genome sequencing

## Abstract

**Background:**

SARS-CoV-2 remains globally circulating, yet post-pandemic comprehensive molecular, serological, and population-level immunity data from low-resource settings such as Ethiopia are limited. This study employed genomic surveillance with seroepidemiological assessment to characterize the epidemiology of SARS-CoV-2 among individuals presenting with influenza-like illness (ILI) and severe acute respiratory infection (SARI) between May 2023 and April 2024 across 21 influenza sentinel surveillance sites representing diverse regions of Ethiopia.

**Methods:**

Respiratory swabs from 8,881 ILI/SARI patients were tested for SARS-CoV-2 by real-time polymerase chain reaction (RT-PCR). Whole-genome sequencing was performed on samples with cycle threshold (Ct) values ≤ 30 using Oxford Nanopore Technology, and serological testing was performed on blood samples collected from participants aged ≥12 years using the WANTAI enzyme-linked immunosorbent assay (ELISA) targeting the spike receptor-binding domain (RBD).

**Results:**

Among 8,881 participants, the overall SARS-CoV-2 detection rate was 3.3 (95% CI: 2.9–3.6). Compared to children under 2 years, older age groups had significantly higher odds of infection: 15–49 years (AOR: 1.60; 95% CI: 1.16–2.21), 50–64 years (AOR: 2.15; 95% CI: 1.31–3.54), and ≥65 years (AOR: 2.37; 95% CI: 1.42–3.95). ILI cases demonstrated higher positivity than SARI cases (AOR: 2.26; 95% CI: 1.75–2.91). SARS-CoV-2 was detected across all seasons, with positivity rates ranging from 1.9% in spring to 6.0% in summer. Whole-genome sequencing of (*n* = 80) revealed exclusive circulation of Omicron lineages, identifying 19 Omicron sub-lineages, predominantly JN.1.18 (31.3%), JN.1 (8.8%), XBB.1.28 (8.8%), BA.2.86 (7.5%), and XBB.1.34.1 (6.3%). Serological analysis of 690 samples demonstrated 96.8% (95% CI: 95.2–98.0%) seropositivity, with no significant demographic variation.

**Conclusions:**

This study demonstrates the continued circulation of SARS-CoV-2 in Ethiopia, primarily among adults with influenza-like illness, with distinct seasonal peaks during the summer. The Omicron variant remained dominant, with JN.1 and its descendants prevailing since late 2023 in parallel with a national rise in COVID-19 cases. These findings reinforce that ongoing genomic and syndromic surveillance utilizing established ILI/SARI platforms remain vital for monitoring viral evolution and guiding targeted public health interventions in low-resource settings.

## Introduction

Severe Acute Respiratory Syndrome Coronavirus-2 (SARS-CoV-2), the causative agent of Coronavirus Disease 2019 (COVID-19), was first reported in Wuhan, China, in December 2019 (1). Globally, the pandemic has resulted in substantial morbidity and mortality (2). Contrary to earlier pandemic predictions, Africa contributed only about 1.2% of global cases and 2.5% of deaths; however, these figures substantially underestimate the true burden due to inadequate diagnostic capacities and weak surveillance and reporting systems (2, 3).

Despite declining global case numbers since mid-2022, SARS-CoV-2 continues to circulate with ongoing variant evolution (3, 4). Persistent transmission has been documented across multiple African settings through influenza-like illness (ILI) and severe acute respiratory illness (SARI) surveillance, with recent SARS-CoV-2 positivity rates of 7.7% in Uganda (5), 10.7% in Kenya (6), 6.7% in Tunisia (7), and 9.2% in Ethiopia (8), underscoring the epidemiological importance of respiratory illness surveillance infrastructure for detecting ongoing transmission in the post-pandemic period.

Understanding population immunity has become critical for assessing disease burden and transmission potential beyond conventional case counts. Data on population immunity from natural infection and vaccination provide insights into the protective capacity of exposed populations (9), with neutralizing antibody levels serving as key predictors of protection against symptomatic infection and severe disease (10). However, the sustained protective capacity of population immunity is fundamentally challenged by rapid antigenic evolution of SARS-CoV-2 and the natural decline of virus-specific antibodies over time. The virus has evolved rapidly, increasing transmissibility and enabling immune evasion, thereby contributing to successive infection waves (3, 11). Multiple variants of concern (VOCs) have emerged, with the Omicron variant and its diverse subvariants exemplifying this threat through high mutation frequency and capacity for immune evasion (2, 3). Together, antibody waning and antigenic escape create a dynamic immunity landscape that cannot be adequately characterized through historical seroprevalence data alone.

Ethiopia reported its first confirmed COVID-19 case on March 13, 2020 (12) and experienced five distinct pandemic waves through 2022 (13). Throughout this period, constrained diagnostic capacity, weak reporting infrastructure, and limited genomic surveillance resulted in substantial underestimation of disease burden. As of July 2025, only 1,088 SARS-CoV-2 sequences from Ethiopia have been deposited in GISAID (14), representing minimal genomic surveillance and substantially constraining understanding of viral evolution and variant circulation patterns in the post-pandemic period.

Ethiopia's vaccination campaign, launched on March 13, 2021, initially targeted healthcare workers and vulnerable populations before expanding to all persons aged ≥12 years by November 2021. Despite approximately 69 million doses administered by December 2023, achieving an estimated 60% coverage among the 73.1 million eligible population, persistent regional disparities in vaccine access and uptake created heterogeneous immunity profiles across Ethiopia's diverse geographic and socioeconomic landscape. A nationwide serosurvey in April–May 2021 reported 10.0% overall seroprevalence, with notable urban-rural differences (urban: 13.0% vs. rural: 8.1%). By late 2021–2022, seroprevalence increased dramatically to 93.0%−99.6% among healthcare workers and urban communities (15), indicating widespread population exposure. However, these estimates represent immunity snapshots from 2021 to 2022 and cannot adequately characterize current population immunity given antibody waning and ongoing viral antigenic evolution.

Comprehensive and recent data on the molecular and serological epidemiology of SARS-CoV-2 in Ethiopia remain scarce, particularly for the post-pandemic period. This study aimed to investigate the molecular and sero-epidemiology of SARS-CoV-2 and associated factors among ILI and SARI cases in Ethiopia during the post-pandemic period, with the goal of characterizing circulating variants, quantifying population immunity, and identifying factors influencing SARS-CoV-2 seroprevalence. Findings will support evidence-based decision-making for appropriate surveillance, vaccination, and intervention strategies.

## Materials and methods

### Study sites, study design, and study period

The influenza sentinel surveillance system in Ethiopia has been operating since 2008. Currently, the system comprises 17 hospitals (SARI) and 4 health centers (ILI). The study employed a multi-center cross-sectional design conducted across 21 government health facilities participating in the ILI/SARI sentinel surveillance program. Data were collected prospectively over a 1-year period, from May 2023 to April 2024.

### Participant enrollment and data collection procedure

Study participants were prospectively enrolled during their visit to the sentinel sites based on World Health Organization (WHO) ILI/SARI case definitions. ILI was defined as an acute respiratory infection (ARI) with a measured temperature of ≥38°C and cough, with symptom onset within the previous 10 days. SARI was defined as ARI with a history of fever or measured fever of ≥38°C, cough, with disease onset within the past 10 days, and requiring hospitalization (16). Participants included patients presenting at sentinel sites who met the WHO ILI or SARI case definitions, provided informed consent (or parental consent for minors with assent for participants aged 12–17 years), and had complete demographic and clinical data. Patients were excluded if they declined to provide consent, were unable to produce adequate respiratory specimens, or had incomplete clinical documentation.

To minimize selection bias, a systematic sampling approach was employed. For ILI surveillance, the first five eligible cases presenting per sentinel site per day were consecutively enrolled. For SARI surveillance, all eligible patients meeting inclusion criteria were enrolled due to the lower number of hospital-based SARI cases during the study period.

For the seroepidemiology analysis, a subset of enrolled ILI and SARI participants aged ≥12 years were invited to provide blood specimen. A sample size of 428 was estimated using the single population proportion formula, assuming a 50% prevalence rate (due to the absence of published data for a similar study in Ethiopia), a 95% confidence level (CL), a 5% margin of error, and a 10% contingency. To enhance statistical power and precision and to optimize resource utilization, a total of 690 participants were ultimately enrolled, proportionally allocated across the sentinel sites based on recent surveillance data.

Standardized data collection forms designed for ILI and SARI surveillance were used by trained personnel to collect demographic, clinical, and epidemiological data. Nasopharyngeal swabs (NP) were collected from each participant and placed in viral transport media (VTM). In addition, blood samples were collected from eligible subsets of ILI and SARI cases. Both the swab and serum samples were transported to the Ethiopian Public Health Institute (EPHI) by trained professionals, at 2–8°C using triple packaging system. Samples were either tested immediately upon receipt or stored briefly at 4°C before testing and then archived at −80°C for long term storage.

### Detection of SARS-CoV-2 by RT-PCR

Viral RNA was extracted using MegaBio plus Virus Purification Kit II (Hangzhou Bioer Technology Co., Ltd., Hangzhou, China) and automated nucleic acid extractor (MGISP-NE32, Wuhan, China) following the manufacturers' instructions. Subsequently, SARS-CoV-2 RT-PCR testing was performed using the Flu-SC2 kit (Cat. No: FluSC2PPB-RUO) or the Flu-SC2-and-RSV multiplex assay (Thermo Fisher Scientific, Cat. No: A47702, Thermo Fisher Scientific, Waltham, United States). Both RT-PCR kits target identical SARS-CoV-2 genes (ORF1ab and N gene) and exhibit comparable analytical sensitivity. The multiplex assay was introduced during the study period to enable simultaneous detection of influenza viruses, SARS-CoV-2, and respiratory syncytial virus (RSV), thereby enhancing surveillance efficiency. As a part of laboratory test quality assurance, internal validation has been conducted at the EPHI and demonstrated ≥98% concordance between the two assays for SARS-CoV-2 detection, ensuring consistency and comparability of results throughout the study period.

### Library preparation and sequencing

SARS-CoV-2 positive samples with the cycle threshold (Ct) of <30 were selected for the genomic sequencing. Complementary DNA (cDNA) was then synthesized using the LunaScript RT Master Mix (New England Biolabs, Ipswich, United States). Briefly, the full SARS-CoV-2 genome was amplified via a tiled amplicon approach following the Midnight protocol, yielding about 1,200 bp amplicons. Amplicons from pools A and B were first combined per sample, then each sample was barcoded individually using the Oxford Nanopore Rapid Barcoding Kit as per the manufacturer's instructions. Barcoded samples were pooled in equally and purified using magnetic beads (17). The concentration of nucleic acid in the prepared library was measured with Qubit HS dsDNA assay (Thermo Fisher Scientific, Waltham, United States).

Purified libraries were normalized to 800 ng to ensure efficient flow cell binding. Adapters were ligated to the libraries as per standard protocol. A standard MinION flow cell (Oxford Nanopore Technologies (ONT), Oxford, United Kingdom) was primed and loaded with the library. Sequencing was performed on a MinION MK1C (MC-113716, Oxford Nanopore Technologies, Oxford, United Kingdom). Raw data and basecalled reads were retrieved from the sequencing device for downstream analysis. Controls were included in the library preparation and sequencing for quality control purposes.

### Bioinformatics analysis

Basecalling and demultiplexing of raw Nanopore data were performed using Guppy (v6.4.2) integrated in MinKNOW (v22.05.9) on the MinION MK1C. This converted raw signals file (FAST5 files) into basecalled reads (FASTQ format) and sorted samples via their barcodes. The resulting FASTQ reads were analyzed with the EPI2ME (v3.4.5) platform using the wf-artic Nextflow workflow (Version: mrt_9127_v110_revp_14jul2021) and V1200 primer scheme (18) to generate a consensus sequence and evaluate sequence quality. NextClade (v3.15.0) was used for clade assignment, lineage classifications, and detailed sequence quality assessment. Sequences with genome coverage >90% and <3,000 ambiguous nucleotides (N) were retained for downstream analysis. Mutation profile, including nucleotide and amino acid substitutions, insertions, and deletions, was obtained from the Nextclade output.

Consensus SARS-CoV-2 sequences were further processed using the Augur toolkit to generate a phylogenetic tree (19). Briefly, the sequences were filtered using the augur filter to retain only those with matching metadata, ensuring a complete dataset. Multiple sequence alignment was performed using MAFFT (20), via the augur align, against the Wuhan-Hu-1 reference genome (NC_045512.2). A maximum likelihood phylogenetic tree was constructed using augur tree with 1,000 bootstrap replicates to assess branch support (21). Finally, the time-resolved phylogenetic tree and metadata were exported in JSON format using augur export for interactive web visualization in Auspice (https://auspice.us/), enabling the exploration of the phylogenetic relationships and metadata attributes.

### Serological analysis of SARS-CoV-2

Serum samples were analyzed for total anti-SARS-CoV-2 antibodies using the WANTAI SARS-CoV-2 Ab ELISA kit (Cat no: WS-1096, Beijing Wantai Biological Pharmacy Enterprise Co., Ltd., Beijing, China). This assay detects total antibodies (IgM and IgG) directed against the receptor-binding domain (RBD) of the SARS-CoV-2 spike (S) protein. The test employs a cut-off index (COI), calculated as the ratio of the sample optical density (OD) to the cut-off OD, with samples showing a COI ≥1.0 considered positive. Each assay plate included manufacturer-provided positive and negative controls, and results were interpreted in accordance with the manufacturer's instructions (22). The assay has been validated to demonstrate no cross-reactivity with antibodies against other coronaviruses (including seasonal HCoV-OC43, HCoV-HKU1, HCoV-NL63, and HCoV-229E) or common respiratory pathogens, with specificity ranging from 99.2% to 100% in independent validation (23, 24).

### Statistical analysis

Statistical analyses were conducted using IBM Statistical Package for the Social Sciences (SPSS) v29.0, Armonk, United States. Descriptive analysis of socio-demographic and clinical characteristics in relation to COVID-19 test positivity was performed using proportions, frequencies, and graphical summaries. Bivariable logistic regression analysis was initially conducted to screen potential predictors COVID-19 positivity. Variables with *p*-values less than 0.20 were then included as candidates in the multivariable logistic regression model. Results from the multivariable analysis are presented as adjusted odds ratios (AORs) with corresponding 95% confidence intervals (CIs), and statistical significance was determined at *p* < 0.05. Missing data was minimal (<2% for key variables) and were handled using complete case analysis. For the multivariable logistic regression, multicollinearity was assessed using variance inflation factors (VIF), with all VIF values <5 indicating no significant collinearity among predictors. Model fit was evaluated using the Hosmer–Lemeshow goodness-of-fit test, where a *p*-value >0.05 indicated an adequate model fit. In addition, residual diagnostics were performed to verify model assumptions and ensure the robustness of the analytical results.

### Data quality assurance

The data was collected by trained health care professionals at each site and, to ensure data quality, close follow up and supervision was provided by dedicated data managers. Each sentinel site is equipped with refrigerators for appropriate temporary sample storage. Tests were performed at National Influenza Center (NIC) at EPHI which was established in 2008 and recognized by WHO as NIC in 2022. The laboratory is the first COVID-19 testing laboratory in Ethiopia, where the COVID-19 index case was confirmed and equipped with necessary infrastructure, supplies, and expertise.

## Results

### Demographic characteristics of study participants

In our study, a total of 8,881 study participants were enrolled with a mean age of 12 and median age of 2.5 years (IQR: 0.9-18.0). The age distribution was highly right-skewed, with 60.8% of participants being children under 5 years of age (Table 1). This distribution reflects the epidemiology of ILI/SARI, which disproportionately affects young children, consistent with the WHO sentinel surveillance framework that prioritizes pediatric SARI cases for severe disease monitoring. The mean age (12 years) was elevated by a smaller proportion of adult and elderly participants (22.6% aged ≥15 years), who also demonstrated higher SARS-CoV-2 positivity rates compared to younger children. Male participants accounted for 53.7% of the total participants study population. The majority of the study participants were from SARI cases (75.1%) and were children under 5 years of age (60.8%). Study participant enrollment was conducted during all four seasons, with the lowest proportion of cases enrolled in spring (21.2%) and the highest in autumn (29.3%) (Table 1).

**Table 1:** Study participants' demographic characteristics, positivity rate of SARS-CoV-2, and associated factors among ILI and SARI cases in selected health facilities in Ethiopia (May 2023–Apr 2024).

**Description**		**Total tests *n* (%)**	**Positive, *n* (%)**	**COR (95% CI)**	***P*-value**	**AOR (95% CI)**	***P*-value**
Sex	Female	4,113 (46.3)	137 (3.3)	1.05 (0.83–1.32)	0.705		
	Male	4,768 (53.7)	152 (3.2)	Ref			
Age group (Years)	<2	3818 (43.0)	96 (2.5)	Ref			
	2–4	1,585 (17.8)	50 (3.2)	1.26 (0.89–1.79)	0.187	1.07 (0.75–1.53)	0.697
	5–14	1,110(12.5)	34 (3.1)	1.23 (0.82–1.82)	0.316	1.09 (0.73–1.62)	0.693
	15–49	1,581 (17.8)	70 (4.4)	1.80 (1.31–2.46)	<0.001	1.60 (1.16–2.21)	0.004^*^
	50–65	426 (4.8)	20 (4.7)	1.91 (1.17–3.13)	0.010	2.15 (1.31–3.54)	0.003^*^
	≥65	361 (4.1)	19 (5.3)	2.15 (1.30–3.57)	0.003	2.37 (1.42–3.95)	<0.001^*^
Admission Type	ILI	2,210 (24.9)	117 (5.3)	2.11 (1.66–2.69)	<0.001	2.26 (1.75–2.91)	<0.001^*^
	SARI	6,671 (75.1)	172 (2.6)	Ref			
Location	Addis Ababa	3,998 (45.0)	136 (3.4)	1.09 (0.86–1.38)	0.477		
	Regions	4,883 (55.0)	153 (3.1)	Ref			
Season	Autumn (Sep–Nov)	2,600 (29.3)	69 (2.7)	1.05 (0.74–1.50)	0.718	1.05 (0.73–1.50)	0.811
	Summer (Dec–Feb)	2,149 (24.2)	128 (6.0)	2.43(1.77–3.34)	<0.0.001	2.54 (1.84–3.51)	<0.001^*^
	Spring (Mar–May)	1,882 (21.2)	36 (1.9)	0.73 (0.49–1.12)	0.213	0.76 (0.49–1.16)	0.196
	Winter (Jun–Aug)	2,250 (25.3)	56 (2.5)	Ref		Ref	
Symptom duration (days)^*^	≤ 3	5,033 (56.7)	164 (3.3)	1.12 (0.65–1.96)	0.680		
	4–7	3,367 (37.9)	111 (3.3)	1.14 (0.65–2.00)	0.655		
	8–10	481 (5.4)	14 (2.9)	Ref			
Total	8,881 (100.0)	289 (3.3)^**^				

ILI, Influenza-like illness; SARI, Severe acute respiratory illness; COR, Crude Odds Ratio; AOR, adjusted odds ratio, 95% CI, 95% Confidence interval; Symptom duration, Duration in days from date of symptom onset to date of sample collection; N/A, Not applicable; Ref, reference group; ^*^significant association (*p* < 0.05); ^**^Overall SARS-CoV-2 positivity was 3.3% (95% CI: 2.9–3.6).

### Detection of SARS-CoV-2 and associated factors

Among the 8,881 study participants, the overall SARS-CoV-2 positivity rate was 3.3% (95% CI: 2.9–3.6). The positivity rates were similar across genders (3.2% in males vs. 3.3% in females), geographic regions (3.4% in Addis Ababa vs. 3.1% in other regions), and duration since symptom onset (ranging from 2.9% to 3.3%). Gender, region, and symptom duration were not statistically significant in bivariable logistic regression analyses (p > 0.20).

Multiple logistic regression was conducted for selected variables, including age group, case category (ILI vs. SARI), and season. Compared with children under 2 years of age, SARS-CoV-2 positivity was significantly higher among individuals aged 15–49 years (AOR: 1.60, 95% CI: 1.16–2.21, *p* = 0.004), 50–64 years (AOR: 2.15, 95% CI: 1.31–3.54), and ≥65 years (AOR: 2.37, 95% CI: 1.42–3.95). Patients presenting with ILI had more than twice the odds of testing positive compared with those with SARI (AOR: 2.26, 95% CI: 1.75–2.91), consistent with their higher observed positivity rate (5.2% vs. 2.2%, *p* < 0.001). Seasonal variation was also evident, with the highest positivity observed in summer (6.0%), corresponding to more than a 2-fold increase in odds compared with winter (AOR: 2.54, 95% CI: 1.84–3.51) (Table 1).

### Genomic diversity of SARS-CoV-2 omicron variant

Of the total of 289 SARS-CoV-2 RT-PCR positive samples, 124 samples with Ct  <30, were selected for whole-genome sequencing. Quality control filtering excluded 44 of these (35.5%) due to insufficient genome coverage (<90%) or excessive ambiguous nucleotides (>3,000 N). The remaining 80 high-quality sequences had a mean genome coverage of 94.2% (range: 90.1–99.8%). Consensus genomes were generated using the EPI2ME bioinformatics analysis platform (Oxford Nanopore Technologies, ONT) and evaluated using Nextclade quality control metrics. Of the 80 cases having successful sequencing data, 45.0% were females, 47.6% were children under 5 years, and 53.8% were categorized as SARI cases. Most sequences originated from sites in Addis Ababa (58.8%) and were collected during 2024 (65.0%) (Supplementary Table S1).

During the study period, all 80 SARS-CoV-2 genomes were identified as Omicron variants, representing seven distinct Nextstrain clades. The predominant clade was 24A (50.0%, *n* = 40), followed by 22F (21.3%, *n* = 17) and 23I (11.3%, *n* = 9). The remaining clades, 23A, 23D, 23E, and 23C, were detected less frequently accounting for 7.5%, 5.0%, 3.8%, and 1.3% of sequences, respectively.

A total of 19 distinct Pangolin lineages were identified. Overall, the dataset was characterized by a predominance of JN.1-related lineages, particularly JN.1.18, along with notable representation of XBB and BA.2.86 sub-lineages. Within Clade 24A, JN.1.18 predominated (31.3%) followed JN.1 (8.8%). Clade 22F was largely represented by XBB sub-lineages, including XBB.1.28 (8.8%), XBB.1.34.1 (6.3%) and XBB.1.34 (5.0%), while Clade 23I was dominated by BA.2.86 (7.5%). Furthermore, lineages XBB.1.5 and FL.25 accounted for 5.0% each and all the remaining lineages covered 22.3% of the distribution reported in this study (Table 2).

**Table 2:** Nextstrain clade and Pango lineage diversity of SARS-CoV-2 Omicron variant among ILI and SARI cases in selected health facilities in Ethiopia, May 2023 to Apr 2024.

**Nextstrain clade (clade display)**	**Total genomes, *n* (%)**	**Pangolin lineage**	***n* (%), within clade**
22F (XBB)	17 (21.3)	GW.5.1.1	1 (1.3)
		XBB.1.28	7 (8.8)
		XBB.1.34	4(5.0)
		XBB.1.34.1	5 (6.3)
23A (XBB.1.5)	6 (7.5)	XBB.1.5	4(5.0)
		XBB.1.5.28	2(2.5)
23C (CH.1.1)	1 (1.3)	CH.1.1.18	1 (1.3)
23D (XBB.1.9)	4(5.0)	FL.25	4(5.0)
23E (XBB.2.3)	3 (3.8)	GE.1.1	1 (1.3)
		GE.1.2.1	2(2.5)
23I (BA.2.86)	9 (11.3)	BA.2.86	6 (7.5)
		BA.2.86.1	3 (3.8)
24A (JN.1)	40 (50.0)	JN.1	7 (8.8)
		JN.1.1	1 (1.3)
		JN.1.16.1	2 (2.5)
		JN.1.18	25 (31.3)
		JN.1.18.1	2 (2.5)
		JN.1.4.5	2 (2.5)
		LY.1	1 (1.3)

### SARS-CoV-2 omicron variant transmission dynamics

**Figure 1:**
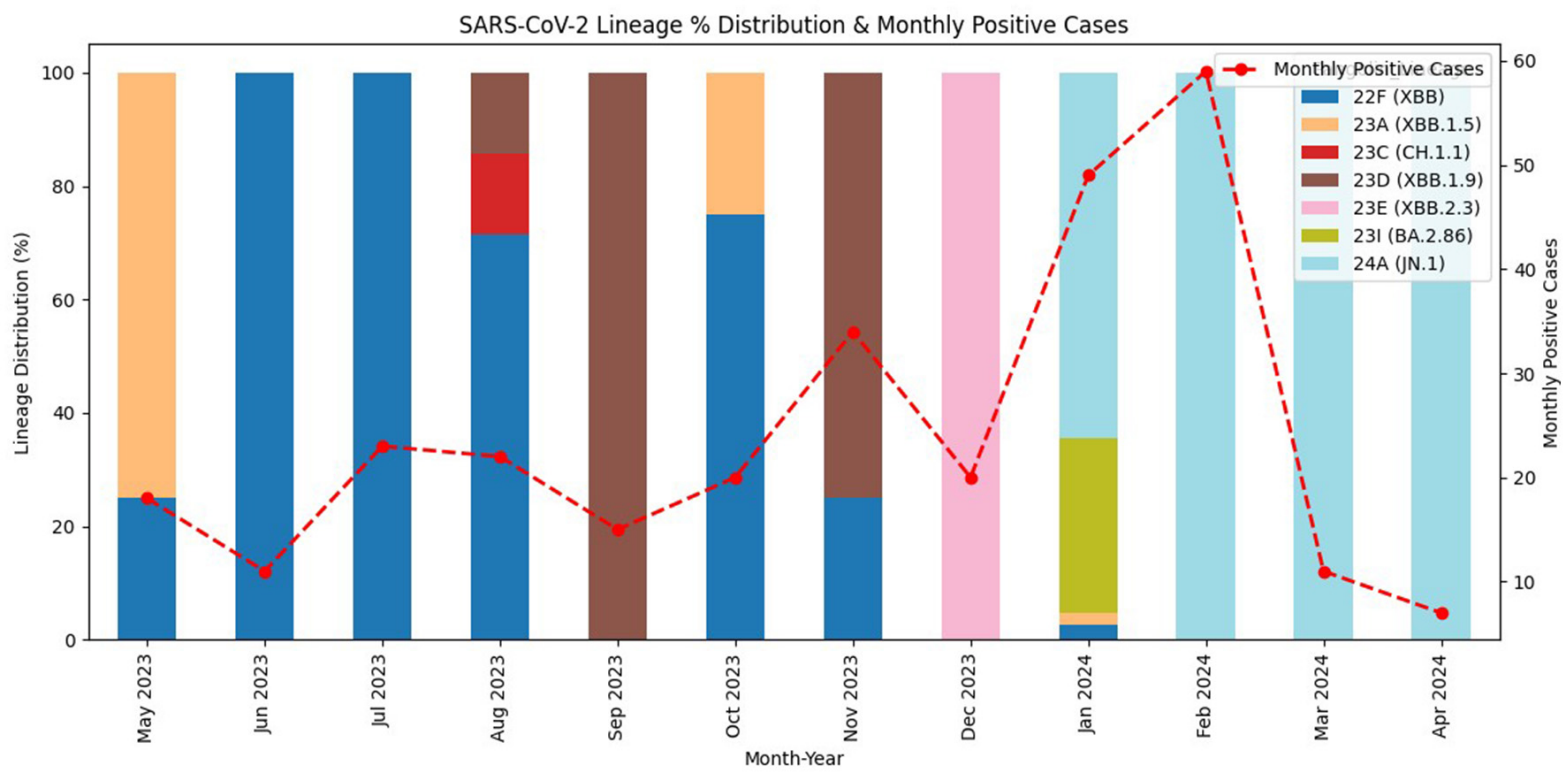
Monthly distribution of SARS-CoV-2 Omicron lineages and corresponding RT-PCR positive cases from May 2023 to April 2024. Stacked bars represent lineage distribution (%) by month, colored according to Nextstrain clade and Pangolin lineage assignment. The red dashed line denotes the monthly number of RT-PCR positive cases. A shift from XBB-related lineages (22F, 23A, 23D, 23E) in mid-2023 to BA.2.86 (23I) in early 2024 was observed, followed by the emergence and subsequent dominance of JN.1 (24A) in 2024, which coincided with the peak in positive cases in February 2024. Data from 8,881 ILI/SARI cases enrolled at 21 sentinel surveillance sites in Ethiopia (study period: May 2023–April 2024). ILI, influenza-like illness; SARI, severe acute respiratory illness; RT-PCR, real-time polymerase chain reaction.

Temporal analysis of SARS-CoV-2 lineages (Figure 1) showed a dynamic shift from May 2023 to April 2024. Clade 22F (XBB lineages) was predominant from May to August 2023. Between September to December 2023, lineage diversity increased, with the emergence of clades 23C (CH.1.1), 23D (XBB.1.9), and 23E (XBB.2.3). In early January 2024, BA.2.86 (Clade 23I) appeared briefly, followed by a rapid transition to JN.1 (clade 24A), which dominated from February through April 2024 (Supplementary Figure S1). The monthly peak in RT-PCR positive cases occurred in February 2024, coinciding with the emergence of JN.1. After this peak, the number of cases declined sharply, although JN.1 remained the predominant circulating variant (Supplementary Figure S2).

### Phylogenetic analysis

Phylogenetic analysis of the 80 SARS-CoV-2 Omicron genomes showed co-circulation of multiple clades in Ethiopia during the study period. This indicates multiple independent introductions followed by localized transmission and sustained community spread. The phylogenetic tree revealed seven distinct clades with most sequences clustering tightly within clade 24A. This clade formed a well-supported monophyletic group characterized by short branch length, consistent with a recent common ancestor. Clade 24A also shared the most recent common ancestor with the second most prevalent clade, 23I (BA.2.86).

**Figure 2:**
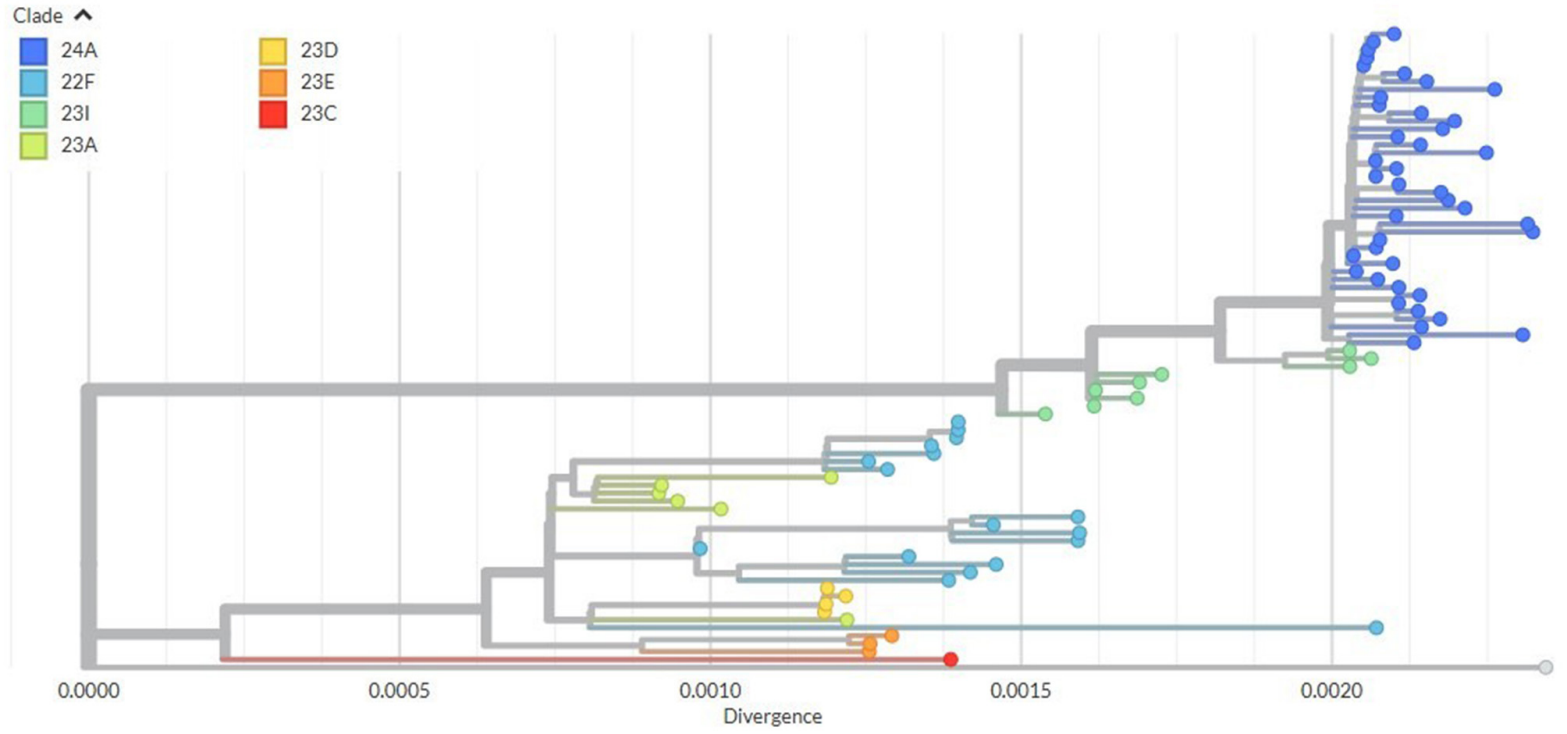
Maximum-likelihood phylogenetic tree of 80 SARS-CoV-2 Omicron variant genome from ILI/SARI cases in Ethiopia (May 2023–April 2024), constructed using Augur with 1,000 bootstrap replicates and visualized in Auspice. Tips are colored according to Nextstrain clade assignment based on the Nextstrain nomenclature system. Bootstrap support values ≥70% are shown at major nodes to indicate confidence levels for key phylogenetic relationships. The x-axis represent the genetic divergence measured as substitutions per site from Wuhan reference genome (*NC_045512.2*), with the corresponding scale bar shown. Data source: 80 whole-genome sequences obtained from ILI/SARI surveillance samples with >90% genome coverage.

In addition to 24A and 23I, other co-circulating clades included 22F, 23A, 23C, and 23D. These clades generally contained fewer sequences, exhibited more scattered distribution and moderate divergence, and showed greater phylogenetic dispersion, suggesting sporadic introductions with limited onward spread and transmission. Sequences were obtained from multiple Ethiopian regions, supporting sustained community transmission across the country (Supplementary Table S1). The overall genetic divergence among sequences ranged from 0.000 to 0.0022 substitutions per site (Figure 2). The phylogenetic analysis also shows distinct clustering of major sub-lineages, with bootstrap values exceeding 70% on the most internal nodes, indicating high confidence in the branching structure (Supplementary Figure S3).

### Seropositivity analysis

Among 690 blood samples tested, 41.0% were from participants aged 12–29-year age group. A higher proportion of samples were obtained from SARI cases (67.2%), from regions outside Addis Ababa (56.2%), from SARS-CoV-2 RT-PCR negative individuals (95.5%), and from unvaccinated participants (72.0%). Overall, the seropositivity rate (anti–SARS-CoV-2 antibodies) was 96.8% (668/690; 95% CI: 95.2-98.0%). Seropositivity was consistently high across subgroups: male: 96.4%, female: 97.3%, a cross age groups (range: 95.5–98.0%), by geographic location (Addis Ababa: 97.0%, regions: 96.6%), by admission type (ILI: 96.9%, SARI: 96.8%), by vaccination status (unvaccinated: 96.6%, vaccinated: 97.3%, unsure: 97.8%), and by PCR status (negative: 96.8%, positive: 96.8%) (Table 3).

**Table 3:** SARS-CoV-2 ELISA test positivity among ILI and SARI cases (*n* = 690) in selected health facilities in Ethiopia, May 2023 to Apr 2024.

**Description**		**Total tests (%)**	**Positive results (%)**
Sex	Male	362 (52.5)	349 (96.4)
	Female	328 (47.5)	319 (97.3)
Age category (years)	12–29	292 (42.3)	282 (96.6)
	30–49	197 (28.6)	193 (98.0)
	50–64	112 (16.2)	108 (96.4)
	≥65	89 (12.9)	85 (95.5)
Admission type	ILI	226 (32.8)	219 (96.9)
	SARI	464 (67.2)	449 (96.8)
Location	Addis Ababa	302 (43.8)	293 (97.0)
	Regions	388 (56.2)	375 (96.6)
SARS-CoV-2 PCR test result	Negative	659 (95.5)	638 (96.8)
	Positive	31 (4.5)	30 (96.8)
Vaccinations status	No	495 (71.7)	478 (96.6)
	Yes	149 (21.6)	145 (97.3)
	Missing	46 (6.7)	45 (97.8)
Total^*^		690 (100.0)	668 (96.8)

ILI, Influenza-like illness; SARI, Severe acute respiratory illness; ^*^96.8%CI (95% CI: 95.2–98.0%).

## Discussion

In this study, we presented a comprehensive finding on the molecular and seroepidemiology of SARS-CoV-2 among ILI and SARI cases in Ethiopia. We found that SARS-CoV-2 was still circulating at a rate of 3.3% among the ILI and SARI cases in Ethiopia in the post pandemic period, which highlights need for health system to remain vigilant by strengthening the surveillance systems and interventions. In this study, age, case classification, and season were significantly associated with SARS-CoV-2 positivity.

The present findings also revealed rapidly evolving and diverse clades and lineages of the Omicron variant circulating in Ethiopia during the post-pandemic period. Omicron clades of 24A (50.0%) and 22F (21.3%) were the most dominant clades, while JN.1.18 (31.0%), JN.1 (8.8%), XBB.1.28 (8.8%), and BA.2.86 (7.5%) were the most prevalent lineages reported in the study. Furthermore, the antibody level among the ILI/SARI cases was high (96.8%), indicating substantial population immunity. Our findings underscores the importance of sustained genomic surveillance for characterizing evolving SARS-CoV-2 variants in post-pandemic settings. The rapidly evolving and diverse Omicron clades and lineages circulating in Ethiopia highlight the need for continuous monitoring and rapid communication of variant data to guide public health decision-making, particularly in relation to vaccination strategies and targeted clinical management approaches.

This research showed that the overall RT-PCR positivity among 8881 cases was 3.3% (95% CI: 2.8–3.6). Similar report of 3.3% positivity rates were reported in Northern Ethiopia (25). However, the report was lower than 9.2%, 7.7%, and 5.7% reported in Ethiopia, Uganda, and China, respectively (5, 8, 26). This difference might be due to different study periods; they were conducted during period of higher transmission periods. Our finding aligns with the global trend showing declining trends of COVID-19 transmission since March 2022, marked by only smaller peaks (3), possibly due to increased population immunity from previous infections and vaccinations (3, 15).

In the current study, a significantly higher SARS-CoV-2 circulation was observed in participants aged ≥15 years. Similar patterns have been reported in China, India, and Europe, where older adolescents and adults showed higher positivity rates (26, 27). Age-stratified analysis indicates persistent adult-predominant transmission in the post-pandemic period, reflecting differential susceptibility and exposure across age groups. The higher positivity observed in older adults may be driven by comorbidities, occupational exposure, or waning immunity following prior vaccination or infection (28). In contrast, lower infection rates among younger children may be associated with reduced Angiotensin-Converting Enzyme 2 (ACE-2) receptor expression (29), and the predominance of other respiratory pathogens such as RSV, which showed a 26.6% positivity rate among children <2 years of age in our study (30). These findings have important implications for targeted surveillance and public health strategies, highlighting the need to prioritize vaccination and preventive measures for older adults and high-risk occupational groups, and to guide age-specific intervention planning (31).

It was further observed that ILI cases had higher SARS-CoV-2 positivity than SARI cases. This finding also align with reports showing that the majority of COVID-19 cases are mild or asymptomatic, with roughly 15% requiring hospitalization (29). Studies in Africa and elsewhere similarly reported that only a small fraction of infections lead to severe disease (3). In fact, a recent surveillance study from China found higher SARS-CoV-2 positivity in ILI than in SARIs, suggesting that many SARS-CoV-2 infections may not progress to hospitalization (26). Furthermore, immunity gained from previous infections and vaccinations has contributed to a reduced disease severity and adverse outcomes (3). Nevertheless, other studies have shown that disease transmission and severity of infection can vary by SARS-CoV-2 variants (32, 33), highlighting the need to monitor disease severity and patient outcomes in relation to circulating variants (34). Moreover, it should be noted that SARI can have other viral, bacterial, or atypical pathogens, requiring more comprehensive laboratory investigations, rather than attributing to SARS-CoV-2 alone.

In our study, SARS-CoV-2 positivity was observed throughout all months of the year, with the highest positivity recorded in January (6.7%) and February (9.7%), followed by a declining trend from March (1.9%) onwards. Minor peaks were also observed during July and November 2023, Table 1 and Supplementary Figure S2. This temporal distribution corresponds to Ethiopia's climatic characteristics, where December–February (locally part of the Bega dry season, corresponding to the southern hemisphere summer) is characterized by dry conditions with cooler temperatures in the highlands and hotter conditions in lowland areas. The transition period (autumn) bridges the main rainy (Kiremt) and dry (Bega) seasons (35). Such dry conditions, particularly when accompanied by cooler highland temperatures, create favorable environments for respiratory virus transmission. This observation is consistent with prior reports from Ethiopia showing that, RSV positivity peaks during during the dry and transitional seasons, autumn (23.8% in autumn and winter 15.0% in winter) compared to summer (30), while chronic respiratory diseases show seasonal morbidity peaks from September to November and June to August, with seasonal indices of 41.47% and 19.16% above the cycle mean, respectively (36). Similarly, WHO has reported that SARS-CoV-2 circulates year-round in Africa and globally, with notable peaks between November and January (37) and continues to circulate with periodic waves in some countries, without a clear seasonal pattern (3). Moreover, studies in Sub-Saharan African countries documented high COVID-19 transmission rates around the months of January and July (38).

The genomic analysis of 80 SARS-CoV-2 samples in the current study revealed that the Omicron variant (100.0%, *n* = 80) was the sole variant detected. The Omicron variant emerged in late 2021, continued to rapidly evolve, and became endemic globally (3, 39). Our analysis revealed that the Omicron variant identified encompasses seven clades further diversifying into 19 lineages. The JN.1 and XBB (22F) and their descendant lineages were predominantly reported, followed by BA.2.86 and XBB.1.5. Similarly, the XBB.1 lineages were also among predominantly reported lineages from Ethiopia in a study conducted in early 2023 (40) in addition to the global (3) and country-specific reports from the USA (41), Pakistan (42) and Morocco (43).

The temporal variant trend in the current study revealed a dynamic shift in SARS-CoV-2 lineage circulation. XBB-derived lineages dominated circulation in mid to late 2023, but were progressively replaced by JN.1 from late 2023 into early 2024. The lineage distribution observed in Ethiopia mirrors global patterns, where XBB-descended lineages prevailed during mid- to late 2023 before being rapidly supplanted by JN.1 subvariants toward the end of 2023 (3). Globally, JN.1, a descendant of BA.2.86, was first detected in August 2023 with key spike mutations conferring enhanced immune evasion and transmissibility advantages. By December 2023 to January 2024, JN.1 had swiftly become the predominant variant across multiple continents, including the USA, Europe, and several Asian nations marking a new phase in post-pandemic SARS-CoV-2 evolution. In the United States, JN.1 rose sharply in late 2023 and maintained predominance through April 2024 (44).

Our findings indicate that Ethiopia experienced the emergence and dominance of the JN.1 lineage concurrent with these global transmission dynamics, highlighting the interconnected nature of SARS-CoV-2 evolution. Despite inherent challenges in real-time genomic surveillance, the variant introduction and replacement patterns in Ethiopia closely mirrored international trends. The temporal overlap between the rise of JN.1 and the marked increase in COVID-19 case numbers in early 2024 (peaking in February) suggests that this lineage substantially contributed to the post-pandemic resurgence during the study period (Supplementary Figure S2).

At the time of analysis, the BA.2.86 and JN.1 sequences generated in this study had not been previously reported or uploaded to GenBank or GISAID from Ethiopia, underscoring a critical gap in national genomic surveillance. Our finding reinforces the need for sustained and regionally coordinated genomic monitoring systems to enable early detection of emerging variants, promote timely data sharing, and facilitate rapid public health response in resource-limited settings. Strengthening platforms such as the ILI/SARI surveillance network will be essential for enhancing data-driven decision-making and maintaining readiness for future SARS-CoV-2 evolutionary events (16).

Phylogenetic analysis of 80 Omicron variant genomes collected in Ethiopia between 2023 and April 2024 revealed seven distinct clades, with the majority of the sequences clustered tightly within clade 24A (JN.1). This monophyletic clustering with short branch length, indicates a recent common ancestor and suggests a rapid local transmission following introductions. Such pattern (short branches length) has been associated with fast spreading variants in a community transmission as reported in other countries during the global expansion of JN.1 in late 2023 and early 2024 (45). Notably, clade 24A (JN.1) shares the common ancestor with clade 23I (BA.2.86), supporting that JN.1 is a descendant of BA.2.86 (39).

In addition to JN.1, we observed the co-circulation of other clades, including 22F (XBB), 23A (XBB.1.5), 23C (CH.1.1), and 23D (XBB.1.9), indicating multiple introductions and subsequent localized transmission of several Omicron sub-lineages. The fact that sequences from multiple regions of Ethiopia, with each region represented by at least one lineage in this study, further supports sustained community transmission nationwide. The limited divergence observed in the current study (0.000–0.0022 substitution per site) reflects the ongoing genomic diversification with low genetic variability among the circulating lineages, likely due to the short evolutionary time frame and dominance of a few successful sub-lineages. This is consistent with prior reports showing that the Omicron variant diversification is driven by both importation and in local transmission (3, 41).

We found a seroprevalence (96.8%) of SARS-CoV-2 antibody among 690 tested ILI/SARI cases, suggesting widespread prior exposure or vaccination in this population. It should be noted that the present study was conducted after extensive COVID-19 vaccination campaigns and after 3 years of the outbreak occurrence in Ethiopia (2) likely contributed to the elevated levels of anti-SARS-CoV-2 antibodies observed. This is further supported by higher antibody levels reported among various population groups during the late and post-pandemic periods in Ethiopia (15, 46). These and other reports highlighted high antibody levels, besides the fear of waning of COVID-19 antibodies over time by other reports (47, 48). On the other hand, high levels of antibody in a community may not always confer cross-protection against the rapidly evolving SARS-CoV-2 variants and sub-lineages (15).

We found a seroprevalence of 96.8% for SARS-CoV-2 antibodies among 690 tested ILI/SARI cases, suggesting widespread prior exposure or vaccination within this population. The exceptionally high seropositivity observed in our study should be interpreted with consideration of potential selection bias, as participants were symptomatic ILI/SARI cases who may have had higher prior SARS-CoV-2 exposure compared to the general population. Nevertheless, this finding aligns with seroprevalence studies conducted in Ethiopia and other settings during the late and post-pandemic periods, reflecting extensive population exposure through both natural infection and vaccination. It should also be noted that this study was conducted following extensive COVID-19 vaccination campaigns and approximately 3 years after the initial outbreak in Ethiopia (2), which likely contributed to the elevated levels of anti-SARS-CoV-2 antibodies observed. As the assay detects anti-spike antibodies, the observed seropositivity represents both infection- and vaccine-induced immunity, and the relative contribution of each source cannot be distinguished using this assay alone. This interpretation is consistent with prior reports documenting high antibody levels among various population groups during the late and post-pandemic periods in Ethiopia (15, 46). Although widespread seropositivity may suggest a high level of population immunity, previous studies have highlighted concerns about waning antibody levels over time (47, 48). Moreover, high antibody titers do not necessarily confer complete cross-protection against the rapidly evolving SARS-CoV-2 variants and sub-lineages (15), emphasizing the continued importance of genomic surveillance and vaccine effectiveness monitoring.

This study has several limitations. The relatively small number of sequenced samples (*n* = 80) compared to the total RT-PCR–positive cases (*n* = 289) may not fully capture the genomic diversity of SARS-CoV-2 circulating in Ethiopia during the study period. Although these sequences represent multiple regions and time points, the limited sampling reduces the generalizability of variant distribution and evolutionary trends. The absence of neutralization assays restricts interpretation of the functional antibody response; while ELISA confirms prior exposure, it does not assess neutralizing capacity or differentiate between infection- and vaccine-induced antibodies. The lack of anti-nucleocapsid testing further limits the ability to differentiate natural infection from vaccination, potentially affecting estimates of infection-derived seropositivity. Logistical and sampling biases may also have influenced the genomic dataset. Delays in sample transport, particularly from remote sentinel sites, could have affected RNA quality and sequencing success, resulting in an overrepresentation of sequences from Addis Ababa and underrepresentation from more remote regions. Incomplete participant information on vaccination status and prior infection history further limits interpretation of the serological findings; as a result, observed seroprevalence reflects overall antibody presence without indicating the source or protective potential of the immune response. Incomplete case capture due to workload fluctuations or limited sentinel site coverage may have affected dataset representativeness. Finally, since the study focused exclusively on symptomatic ILI and SARI cases, asymptomatic and mildly symptomatic SARS-CoV-2 infections were not assessed, meaning that overall community-level viral circulation may have been underestimated.

## Conclusions

This study revealed that SARS-CoV-2 circulated year-round in Ethiopia from May 2023 to April 2024, with an overall positivity rate of 3.3%. Higher detection rates were observed among individuals aged over 15 years and among ILI cases, with a pronounced peak in cases during the summer (dry) season. Seven Omicron clades comprising 19 diverse lineages were detected, with JN.1.18, JN.1, XBB.1.28, and BA.2.86 emerging as the predominant circulating lineages, each exhibiting varying dominance across the study period. XBB lineages were present from mid-2023 until January 2024 and were progressively replaced by JN.1 in early 2024. The predominance of JN.1 coincided with an increase in the number of COVID-19 cases, suggesting it was a major driver of transmission at that time.

The detection of diverse and rapidly evolving Omicron lineages highlights the importance of ongoing SARS-CoV-2 variant monitoring and underscores the value of combining genomic surveillance with syndromic surveillance using the ILI/SARI system to guide public health interventions and mitigate the impact of COVID-19. High levels of anti-SARS-CoV-2 antibodies observed in this study suggest considerable population immunity; however, this finding should be interpreted with caution, as antibody levels do not necessarily equate to complete protection against infection, especially given the evolving genomic landscape. Overall, these findings demonstrate the feasibility and public health benefit of integrating genomic surveillance into existing ILI/SARI sentinel platforms in low-resource African settings.

## Data Availability

The datasets presented in this study can be found in online repositories. The names of the repository/repositories and accession number(s) can be found in the article/Supplementary material.
