# Supplementary material for: Molecular and seroepidemiology of SARS-CoV-2 among influenza-like illness and severe Acute respiratory illness cases in selected health facilities in Ethiopia
Source: Front Public Health. 2026 Jan 26;13:1714899. doi: 10.3389/fpubh.2025.1714899 (PMC12883655; doi:10.3389/fpubh.2025.1714899)
Supplement: Supplementary file 1 [file Supplementary_file_1.docx]

Supplementary Material

# Supplementary Figures and Tables

## Supplementary Table

**Supplementary Table S1**: Number of SARS-CoV-2 sequenced samples among ILI/SARI cases in selected sites in Ethiopia, May 2023 to April 2024

| **Description** | | | **Frequency** | **Percentage** |
| --- | --- | --- | --- | --- |
| Sex | Female | | 36 | 45.0 |
|  | Male | | 44 | 55.0 |
| Age Category (Years) | <2Years | | 29 | 36.3 |
|  | 2-<5 | | 9 | 11.3 |
|  | 5-<15 | | 7 | 8.8 |
|  | 15-<50 | | 21 | 26.3 |
|  | 50-<65 | | 9 | 11.3 |
|  | 65+ | | 5 | 6.3 |
| Case Category | ILI | | 37 | 46.3 |
|  | SARI | | 43 | 53.8 |
| Sites and Region of Case enrolment | **Site** | **Region** |  | |
|  | Akaki Health Center | Addis Ababa | 17 | 21.3 |
|  | Dilfire Health Center | Addis Ababa | 4 | 5.0 |
|  | Kolfe Health Center | Addis Ababa | 5 | 6.3 |
|  | Shiromeda Health Center | Addis Ababa | 11 | 13.8 |
|  | St. Peter Hospital | Addis Ababa | 6 | 7.5 |
|  | Yekatit 12 Hospital | Addis Ababa | 2 | 2.5 |
|  | Zewditu Memorial Hospital | Addis Ababa | 2 | 2.5 |
|  | Adama Teaching Hospital | Oromia Region | 4 | 5.0 |
|  | Adare General Hospital | Sidama Region | 4 | 5.0 |
|  | Arbaminch General Hospital | Central Ethiopia | 1 | 1.3 |
|  | Asosa Hospital | Benishangul-Gumuz | 3 | 3.8 |
|  | Butajira General Hospital | Central Ethiopia | 1 | 1.3 |
|  | Dilchora Hospital | Dire Dawa | 9 | 11.3 |
|  | Dubti Hospital | Afar | 1 | 1.3 |
|  | Felgehiwot Hospital | Amhara | 3 | 3.8 |
|  | Gambella Hospital | Gambella | 2 | 2.5 |
|  | Jinka General Hospital | South Ethiopia | 1 | 1.3 |
|  | Mekele general Hospital | Tigray | 2 | 2.5 |
|  | Shanangibe Hospital | Oromia | 2 | 2.5 |
| Year of sample collection | 2023 | | 28 | 35.0 |
|  | 2024 | | 52 | 65.0 |

Key: ILI: Influenza like illness; SARI: Severe acute respiratory illness

## Supplementary Figures


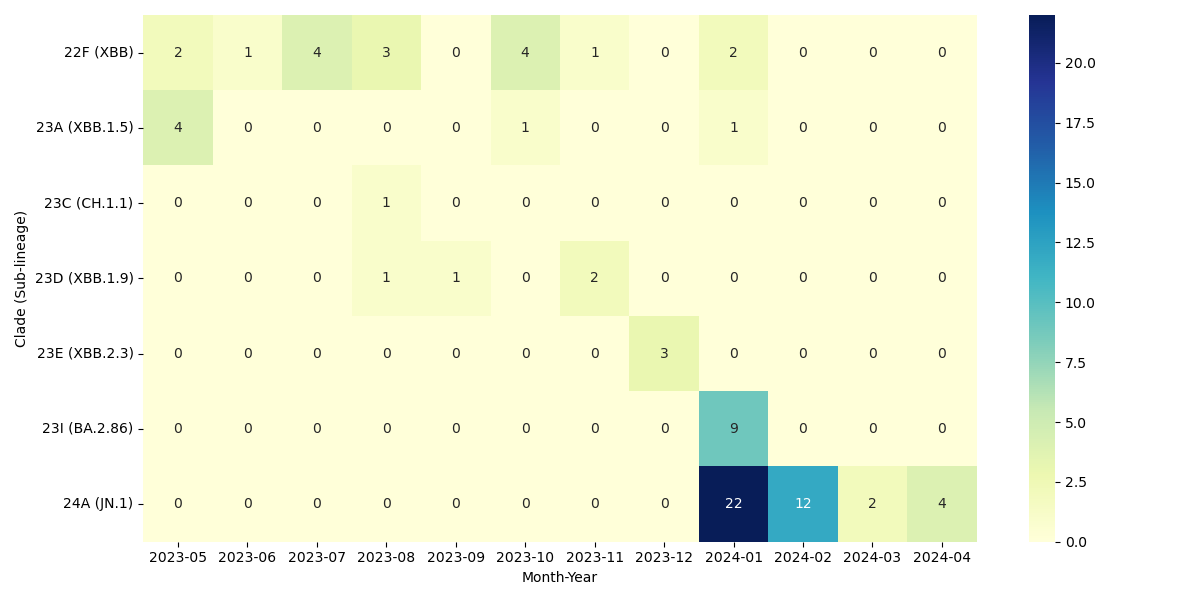
 **Supplementary Figure S1**. Heatmap showing the monthly absolute frequency of Clade or Omicron sub-lineages across time, with color intensity representing proportional detection frequency per month

**Supplementary Figure S2:** Monthly SARS-CoV-2 ILI/SARI case enrolment and positivity trends among ILI/SARI cases in selected health facilities in Ethiopia, May 2023-Apr 2024. The numbeers in the brackets the SARS-CoV-2 positivity rates per the specified month


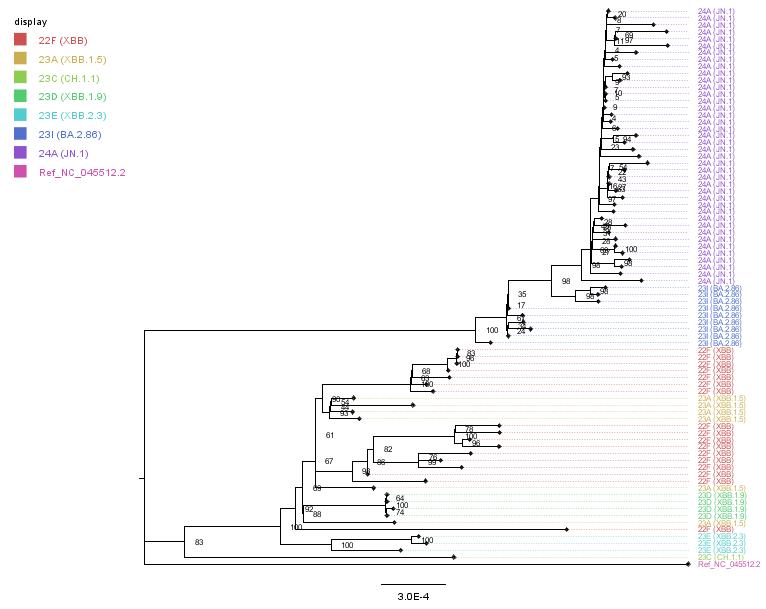
**Supplement Figure S3**: Maximum likelihood tree inferred from whole-genome sequences and the numbers at nodes indicate bootstrap support values from 1,000 replicates. Scale bar represents substitutions per site.
